# Long-term evaluation of intraoperative neurophysiological monitoring-assisted tethered cord surgery

**DOI:** 10.1007/s00381-017-3478-y

**Published:** 2017-07-04

**Authors:** S. E. Dulfer, G. Drost, F. Lange, H. L. Journee, F. H. Wapstra, E. W. Hoving

**Affiliations:** 1 Department of Neurosurgery, University Medical Centre Groningen, University of Groningen, PO Box 30.001, 9700RB Groningen, The Netherlands; 2 Department of Neurology, University Medical Centre Groningen, University of Groningen, PO Box 30.001, 9700RB Groningen, The Netherlands; 3 Department of Orthopedics, University Medical Centre Groningen, University of Groningen, PO Box 30.001, 9700RB Groningen, The Netherlands

**Keywords:** Tethered spinal cord surgery, Intra-operative neurophysiological monitoring, Follow-up, Lipomyelomeningocele, Split cord malformation, Myelomeningocele

## Abstract

**Purpose:**

Patients with tethered spinal cord have been investigated for short-term effects after tethered spinal cord surgery in the past. However, little is known about the long-term effects in this patient group. In this retrospective, longitudinal, observational study, a patient sample of a previous report of 65 patients was reassessed to observe the long-term effects of intraoperative neurophysiological monitoring-assisted tethered cord surgery.

**Methods:**

With the use of patient charts and a survey, patients were scored on four domains: (1) neurological deficits, (2) urological deficits, (3) pain symptoms, and (4) orthopedic deficits. Measurements were performed at four moments in time: (1) preoperatively, (2) postoperatively, (3) follow-up 1 (4.6 years), and (4) follow-up 2 (11.2 years). Besides this, a subgroup analysis and a quality of life questionnaire were performed.

**Results:**

When observing the symptom domains in the long-term, the pain domain appeared to improve most postoperatively after which it remained stable over time. The neurological and urological domains showed a stable, slightly decreasing trend in the long-term follow-up. The orthopedic domain showed a significant increase of the number of patients with scoliosis during the long-term follow-up.

**Conclusions:**

Lasting effects of stability in the neurological, urological, and pain domains were observed. Close monitoring during follow-up might contribute to early recognition of progressive scoliosis, in spite of detethering, in a risk group defined by females who underwent tethered cord surgery at or under the age of 12 years old with either lipomyelomeningocele, split cord malformation, or myelomeningocele. Detethering does not appear to protect these patients against progressive scoliosis.

## Introduction

Spinal dysraphism refers to a large variety of congenital malformations, including lipomyelomeningocele, split cord malformation, myelomeningocele, tight filum terminale, and dermoid sinus [14]. As soon as a patient with a tethered cord morphology becomes symptomatic one speaks of a tethered cord syndrome [21, 42]. The symptoms associated with tethered cord syndrome may affect four different fields: (1) neurological, (2) urological, (3) pain, and (4) orthopedic. Neurological symptoms appear as sensory and/or motor loss of the caudal nerve roots involved. Urological disturbances affect bladder function. This may start subtle, and urodynamic investigation is used for diagnosis. Orthopedical symptoms may express as structural deformations like a clubfoot or progressive scoliosis. Pain can be atypical without the classical features of radicular or neurogenic pain. The presence of these symptoms determines the indication for surgical detethering, although prophylactic surgery is recommended in asymptomatic young children with lipomyelomeningocele [4, 12, 36, 38]. The assessment of symptoms pre- and postoperatively helps to define the outcome of the surgery, but the evaluation of the quality of the surgical detethering appears to be more complicated [13]. The distinction between symptoms due to tethering versus myelodysplasia cannot always be made, and the reversibility of symptomatic tethering is also related to the timing of surgical release. These aspects complicate the evaluation of the use of intraoperative neurophysiological monitoring (IONM) in tethered cord surgery. IONM has been widely used as a tool to improve surgical results concerning safety (prevention of neurological morbidity) and efficacy (lasting effect of detethering) [17, 18, 21, 28, 32, 39].

In IONM, we can distinguish between mapping and monitoring. Mapping refers to identification of functional nerve roots by means of direct nerve root stimulation [35]. In the same manner, non-functional tethering structures can be identified and safely cut. Monitoring provides continuous information about the preservation of the motor and/or sensory pathways, and in this way, it may serve as a reassurance for the neurosurgeon during the process of surgical detethering [35]. Besides mapping, motor evoked potentials, sensory evoked potentials, or bulbocavernosus reflexes are being used for monitoring in tethered cord surgery, although the clinical relevance is unknown yet [35].

In our previous report on a series of 65 patients with tethered cord based on spinal dysraphism, that had been surgically detethered with the use of IONM, we concluded that the use of IONM, both mapping and monitoring, appeared to be feasible in all patients irrespective of age, and that its use might have contributed to the prevention of preoperative neurological and urological morbidity in the high-risk group [13]. This high-risk group consisted of patients with lipomyelomeningoceles [29] or with split cord malformations type I [26], while the higher risk profile referred to the increased risk of surgical morbidity in these patients. The surgical strategy in lipomyelomeningoceles at that time was to perform a maximal resection of the lipoma in addition to creating a maximal safe detethering. Lipomas were not resected radically, and a small remnant was considered acceptable. The mean follow-up (FU) in this series was 4.2 years, and the secondary progression of symptoms was described.

An additional indication of effective surgical detethering might be concluded from the long-term evaluation of these patients. The duration of stabilization of symptoms versus recurrence of identical symptoms or appearance of new symptoms might be related to the surgical detethering. This study presents the long-term FU results of the same cohort of patients that have been reported on before [13].

## Method

### Population

**Fig. 1:**
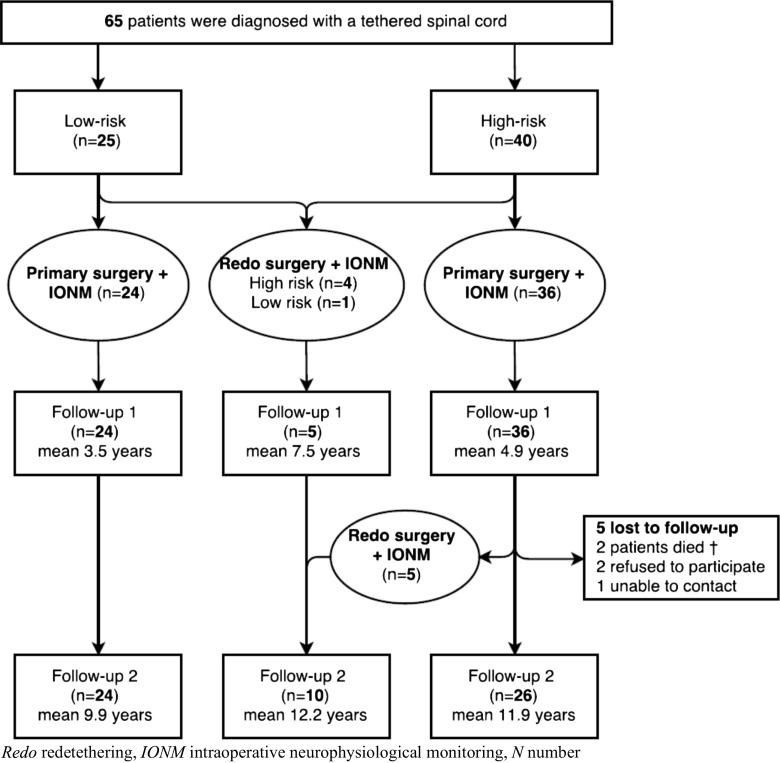
Flowchart patient population

A previously described cohort of 65 patients, that underwent IONM-assisted tethered cord surgery, was reevaluated to asses a long-term FU. Patients were scored at four moments in time: (1) preoperatively, (2) postoperatively, (3) at follow-up 1 (mean 4.6 years), and (4) at follow-up 2 (mean 11.2 years). A distinction was made between a low-risk group (LRG), consisting of 25 patients, and a high-risk group (HRG) of 40 patients (Fig. 1). The risk profile was determined by the surgical neurological morbidity related to specific dysraphic morphologies as known from literature [13, 27, 29]. High-risk patients comprised the diagnosis of lipomyelomeningocele and split cord malformation type I. The other diagnoses were considered to belong to the low-risk group. Twenty-four patients of the LRG underwent primary tethered spinal cord surgery, and one patient underwent a redetethering (redo). Of the HRG, 36 patients underwent primary tethered spinal cord surgery, and four patients underwent a redo. A first FU (FU1) with a mean of 4.6 years has been performed in the past. The present study has performed a second FU (FU2), with a mean of 11.2 years. Between FU1 and FU2, five patients were lost to FU, and five patients of the HRG underwent a redo. At FU2, the LRG consisted of 24 patients, the HRG of 36 patients, and a redo group of 10 patients, of which nine patients (90%) belonged to the HRG.

### Symptom domains

In this article, an overall view of 60 patients at four moments of clinical evaluation was obtained. Also, a per-patient analysis was made comparing postoperative outcome with FU2 outcome. Pre-operative, postoperative, and FU1 scoring has been described in our earlier report [13], and accordingly scoring was performed on four domains at FU2—neurological deficits, urological deficits, pain symptoms, and orthopedic deficits (Table 1). The scoring values ranged from 0 to 3 (0 asymptomatic, 1 minimal, 2 moderate, and 3 severe). In comparing scores at the different moments of clinical evaluation, patients could have improved or deteriorated with 1, 2, or 3 steps, or remained stable.

**Table 1:** Scoring of deficits [13]

Deficit	Neurological	Urological	Pain	Orthopedic
0 = asymptomatic	–	–	–	–
1 = minimal	Sensibility	VU abnormal	Minimal pain	Minimal scoliosis
2 = moderate	Motor minimal (≥4)	Bladder residue	Moderate pain	Moderate scoliosis
3 = severe	Motor paresis (<4)	Incontinence	Severe pain	Severe scoliosis

*VU* video urodynamics

Additional information at FU2 was obtained by investigating the patient charts and by performing a survey administered by telephone to all the patients. Concerning pain, a questionnaire with a validated visual analogue scale (VAS) ranging from 0 to 10, was used. Scoliosis was scored based on Cobb angle measurements which were checked by an orthopedic surgeon (FW). When there was no x-ray available, magnetic resonance imaging (MRI) was used. Cobb angles varied from 5° to 20° (minimal scoliosis), 21° to 40° (moderate scoliosis), and >40° (severe scoliosis); only an increase of >5° was considered as progression.

### Subgroup analysis

A subgroup analysis was performed for the patients diagnosed with a lipomyelomeningocele and the patients diagnosed with a tight filum terminale. The progression-free survival (PFS) after surgery at FU2 was analyzed within this subgroup analysis as well.

### Quality of life

A quality of life questionnaire, the Medical Outcome Study 36-item Short-Form Health Survey (SF-36), was performed to assess the long-term quality of life after IONM-assisted tethered cord surgery. The SF-36 consists of 36 questions, divided into eight scales: physical functioning, role limitations caused by physical health problems, bodily pain, general health perceptions, vitality, social functioning, role limitations caused by emotional problems, and general mental health. All scales are ranged from 0 to 100 in which a higher score is equal to a better health status. Since the SF-36 is validated from the age of 16, our population was divided into two groups: tethered cord (TC) children (<16 years, *n* = 18) and TC adults (≥16 years, *n* = 37). We compared the results of these two age groups and our total population with a healthy Dutch population (*n* = 1742) [1]. Our series was also compared with other series consisting of a myelomeningocele population [2], a spina bifida occulta population [40], and again with the healthy Dutch population.

### Intraoperative neurophysiological monitoring

IONM was used in a standardized fashion, using both mapping and monitoring. Mapping was performed by direct monopolar nerve root stimulation in order to distinguish between functional nerve roots and other tethering structures. Monitoring was used by means of transcranial electrical motor evoked potential stimulation in order to assess the integrity of the motor pathways. Bulbocavernosus reflexes and sensory evoked potentials were not used in a standard fashion since this technique was being developed at that time. IONM methods have been previously described [13].

## Results

### Symptom domains

**Fig. 2:**
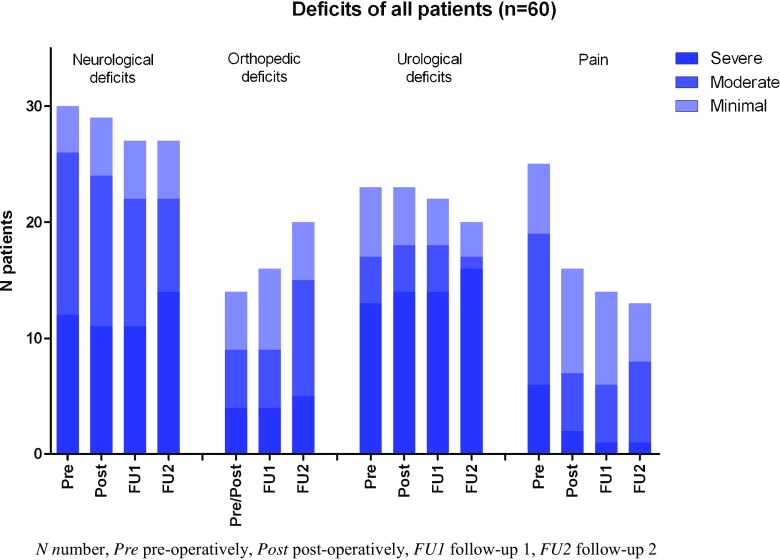
Number of patients with deficits per symptom domain per measure moment (*n* = 60). The neurological and urological domains showed a small decrease over time. The orthopedic domain showed an increase in the number of patients and the severity of the symptoms over time. Pain symptoms decreased directly postoperatively after which a stable trend was observed

Figure 2 presents an overall view of the number of symptomatic patients per domain at the four moments of clinical evaluation: pre- and postoperatively, at follow-up 1, and at follow-up 2. Neurologically, overall, a small decrease in the number of symptomatic patients was observed. Urologically, a similar trend was seen. Pain showed a decrease in the number of symptomatic patients directly postoperative. This stabilized during follow-up. The orthopedic domain showed a gradual increase in the number of symptomatic patients.

A per-patient analysis was performed comparing postoperative outcome with FU2 outcome. The changes in outcome are described below per symptom domain and are shown in Table 3 in the appendix.

#### Neurological

In the neurological domain, six patients (10.0%) improved. Of these, three patients belonged to the HRG. One patient improved with a single step and two with two steps. The remaining three patients belonged to the LRG, of which two had improved with a single step and one with two steps. Of the redo’s, no patients had improved. Seven patients (11.7%) deteriorated, of which three belonged to the HRG. Two patients deteriorated with a single step and one patient with two steps. In both the LRG and the redo group, two patients had a single step deterioration.

#### Urological

Urologically, seven patients (11.7%) had improved, of which five patients in the HRG: two patients with one step, two patients with two steps, and one patient with three steps. In the redo group, no patients had improved. Four patients (6.7%) deteriorated, of which one patient in the HRG with three steps from no urological symptoms to incontinence. Two patients deteriorated in the LRG, of which one patient with one step and one patient with three steps. In the redo group, one patient deteriorated with three steps.

#### Pain

Pain symptoms improved in eight patients (13.3%), of which two in the HRG with one step. The same applied for the LRG. In the redo group, three patients improved with one step, and one patient improved from severe pain to no pain (three steps). Four patients (6.7%) deteriorated, of which two patients with two steps in the HRG and two patients with one step in the LRG.

#### Orthopedic

In the orthopedic domain, data could be obtained of 59 patients due to the data of one patient missing. An improvement of one step for scoliosis was seen in one patient in the redo group. Eleven patients (18.6%) deteriorated which means they had progressive scoliosis. Seven of these patients belonged to the HRG, of which four patients deteriorated with one step and three patients with two steps. In the LRG, three patients deteriorated, of which one patient with one step and two patients with two steps. From the redo’s, one patient deteriorated with one step.

Five patients deteriorated between postoperative measurements and FU1. The remaining six patients deteriorated between FU1 and FU2. Table 2 shows the baseline characteristics of the 11 patients that had progressive scoliosis. The mean age at surgery was 4.6 years (standard deviation (SD) 4.3 years, min–max 0.1–12.1 years). Ten patients (90.9%) were female, and seven patients (63.6%) belonged to the HRG. Two patients that belonged to the LRG were diagnosed with myelomeningocele, and one patient was diagnosed with tight filum terminale. Two patients had syringomyelia, two others had an intramedullary cyst, and one patient had both. One patient belonged to the redo group. In total, 19 females underwent surgery with an age at or younger than 12 years, of which five had scoliosis postoperatively, and 13 females had scoliosis at FU2. Thirteen males underwent surgery with an age at or younger than 12 years, of which one patient had missing data in the orthopedic domain. Of the remaining 12 patients, one had scoliosis postoperatively and at FU2, although his scoliosis changed from minimal scoliosis to moderate scoliosis.

**Table 2:** Baseline characteristics patients with progressive scoliosis

	Age of surgery (years)	Age at FU2 (years)	Sex	Risk group	Diagnosis	Comorbidity
Patient 1	1.1	14.1	Female	HRG	SCM + TFT	–
Patient 2	12.1	24.8	Female	HRG	LMC + SCM + TFT	–
Patient 3	1.1	13.8	Female	HRG	LMC	Redo during FU
Patient 4	6.8	19.5	Female	Redo	LMC + TFT	–
Patient 5	1.0	16.4	Female	HRG	SCM + TFT + dermoid sinus	Intramedullary cyst
Patient 6	3.6	18.3	Female	HRG	LMC + TFT	Syringomyelia
Patient 7	1.0	13.6	Female	HRG	LMC	Syringomyelia
Patient 8	6.1	17.2	Female	HRG	SCM + TFT	–
Patient 9	6.7	14.6	Female	LRG	TFT	Intramedullary cyst
Patient 10	11.3	20.1	Female	LRG	MMC	–
Patient 11	0.1	9.2	Male	LRG	MMC	Syringomyelia + intramedullary cyst
Mean (SD)	4.6 (4.3)	16.5 (4.2)				
%			91% female	73% HRG		

*HRG* high-risk group, *LRG* low-risk group, *Redo* redetethering group, *FU* follow-up, *SCM* split cord malformation, *TFT* tight filum terminale, *LMC* lypomyelomeningocele, *SD* standard deviation

In four patients (36.4%), no baseline x-ray was available. Three of them had no scoliosis at the pre/postoperative moment of measuring, and for the other patient, an MRI was used to measure the Cobb angle.

Mean time between pre/postoperative spine x-rays and FU2 spine x-rays was 6.2 years (SD 2.4 years).

### Subgroup analysis

#### Lipomyelomeningocele

**Fig. 3:**
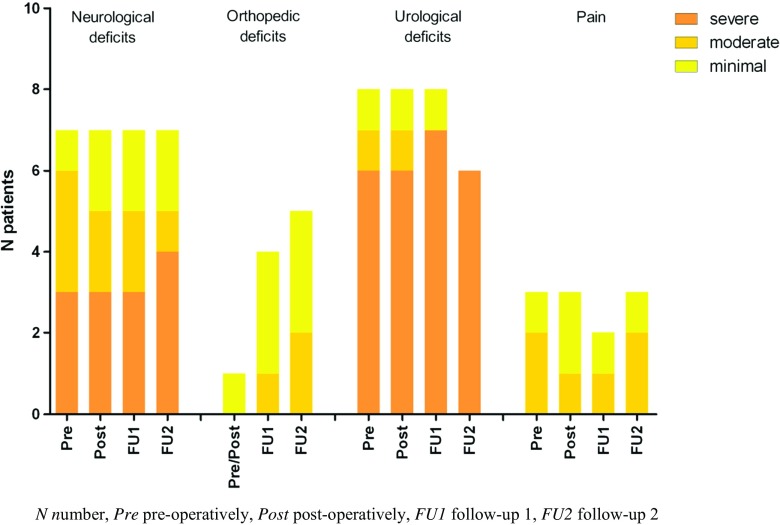
Number of lipomyelomeningocele patients with deficits per symptom domain per measure moment (*n* = 14). The neurological domain remained stable over time with slight changes of severity. The urological domain showed a small decrease after FU1, and the pain domain remained near stable. The orthopedic domain showed an increase in patients with scoliosis and the severity of their scoliosis

Fourteen patients diagnosed with lipomyelomeningocele underwent primary surgery with IONM. Six patients were asymptomatic, and eight patients were symptomatic before surgery. All lipomyelomeningoceles were resected partially. The PFS after surgery was 64.3% after a FU of 12.4 years. The asymptomatic patients had a PFS of 50.0% after a FU of 11.6 years, and the symptomatic patients had a PFS of 75.0% after a FU of 12.9 years. In Fig. 3, an overview can be seen of the number of patients with deficits per domain over time. The neurological, urological, and pain domain showed a stable outcome over time with slight changes of severity. The orthopedic domain however showed a 35.7% increase of patients with progressive scoliosis.

#### Tight filum terminale

The criteria for resecting a tight filum terminale were a tethered cord syndrome and the presence of a thickened filum terminale in combination with a low-lying conus [34]. Ten patients met these criteria and were diagnosed with a tight filum terminale as single abnormality. The PFS after surgery was 80.0% after a FU of 10.5 years.

**Fig. 4:**
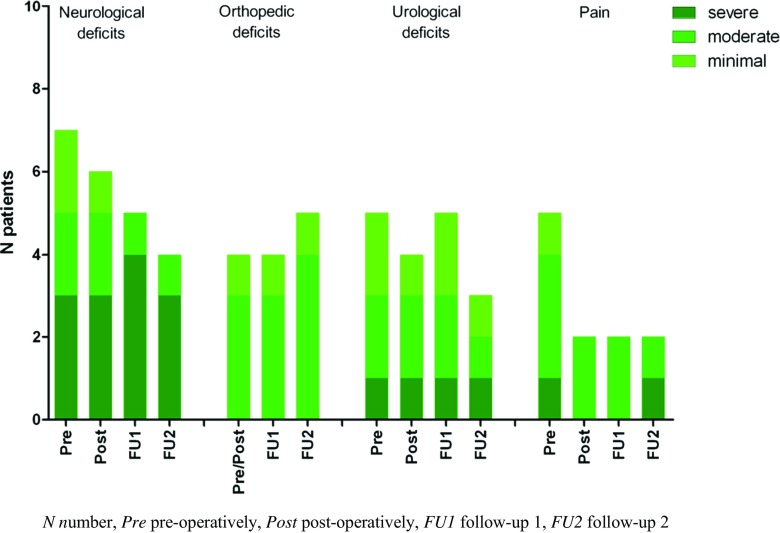
Number of tight filum terminale patients with deficits per symptom per measure moment (*n* = 10). The neurological domain showed a decrease, over time, in the number of patients with neurological symptoms. The orthopedic and urological domains showed slight differences in the number of patients with deficits over time. The pain domain showed a decrease directly postoperative

When comparing post and FU2 results, the orthopedic, urological, and pain domains show a stable outcome (Fig. 4). A decrease of the number of patients with neurological deficits was observed.

### Quality of life

**Fig. 5:**
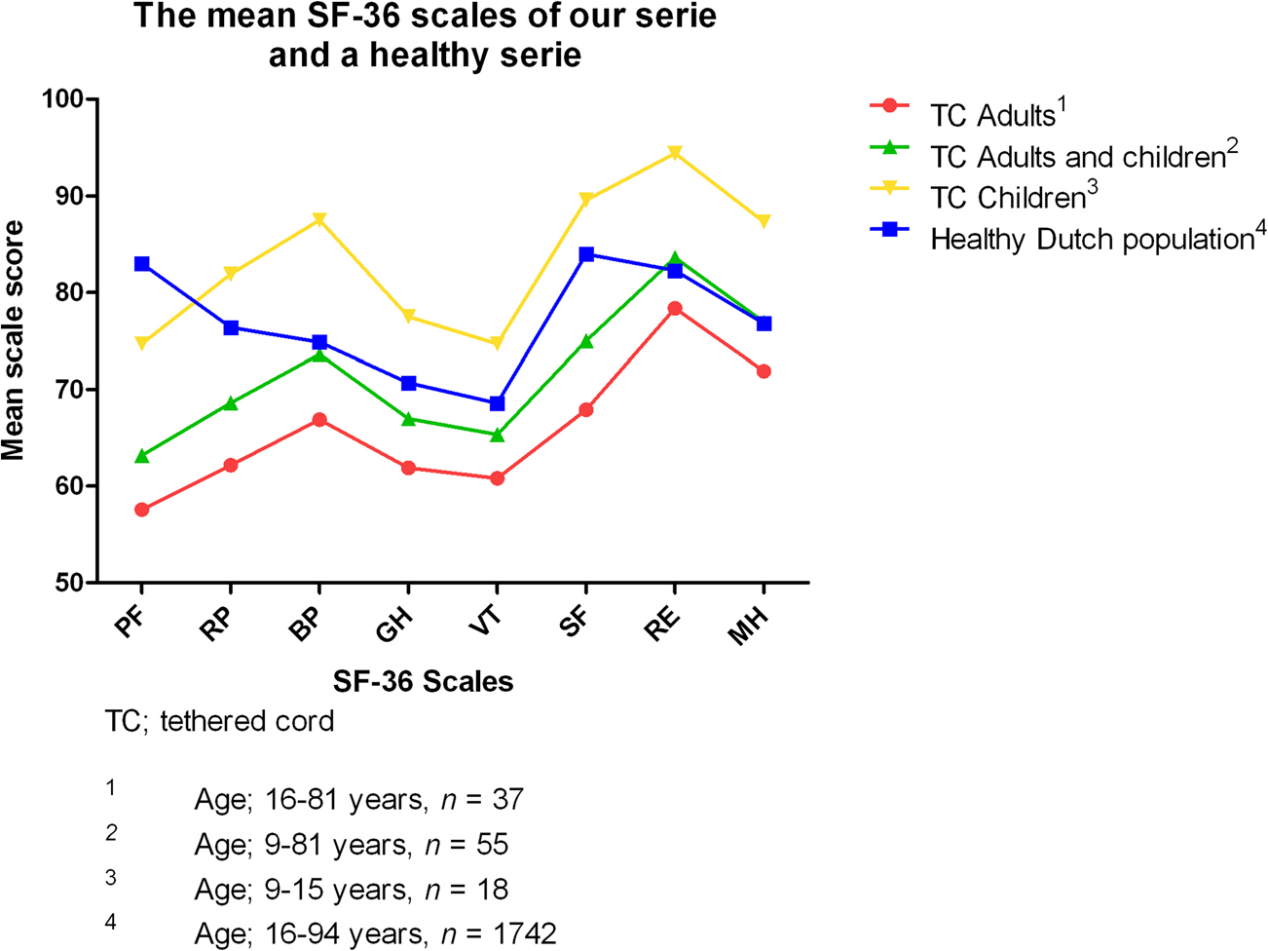
Mean SF-36 scales of our series and a healthy Dutch population. The TC adults scored lower on every domain when compared with the Dutch population [1]. The TC children scored higher on every domain except the PF domain. *TC* tethered cord, *PF* physical functioning, *RP* role limitations caused by physical health problems, *BP* bodily pain, *GH* general health perceptions, *VT* vitality, *SF* social functioning, *RE* role limitations caused by emotional problems, *MH* general mental health

The SF-36 was filled in by 55 patients (91.7%). Six out of eight scales (physical functioning, role limitations caused by physical health problems, bodily pain, general health perceptions, vitality, and social functioning) were significantly different (*p* > 0.05) when comparing the TC adults group with the healthy Dutch population. The scales role limitations caused by emotional problems and general mental health were not significantly different with a *p* value of 0.48 and 0.09, respectively. Figure 5 shows the mean scores per scale of the different age groups and of the healthy Dutch population. The TC children have a higher mean score for every scale except physical functioning when compared to the healthy Dutch population. The TC adults have a lower mean score in every domain than the healthy Dutch population.

**Fig. 6:**
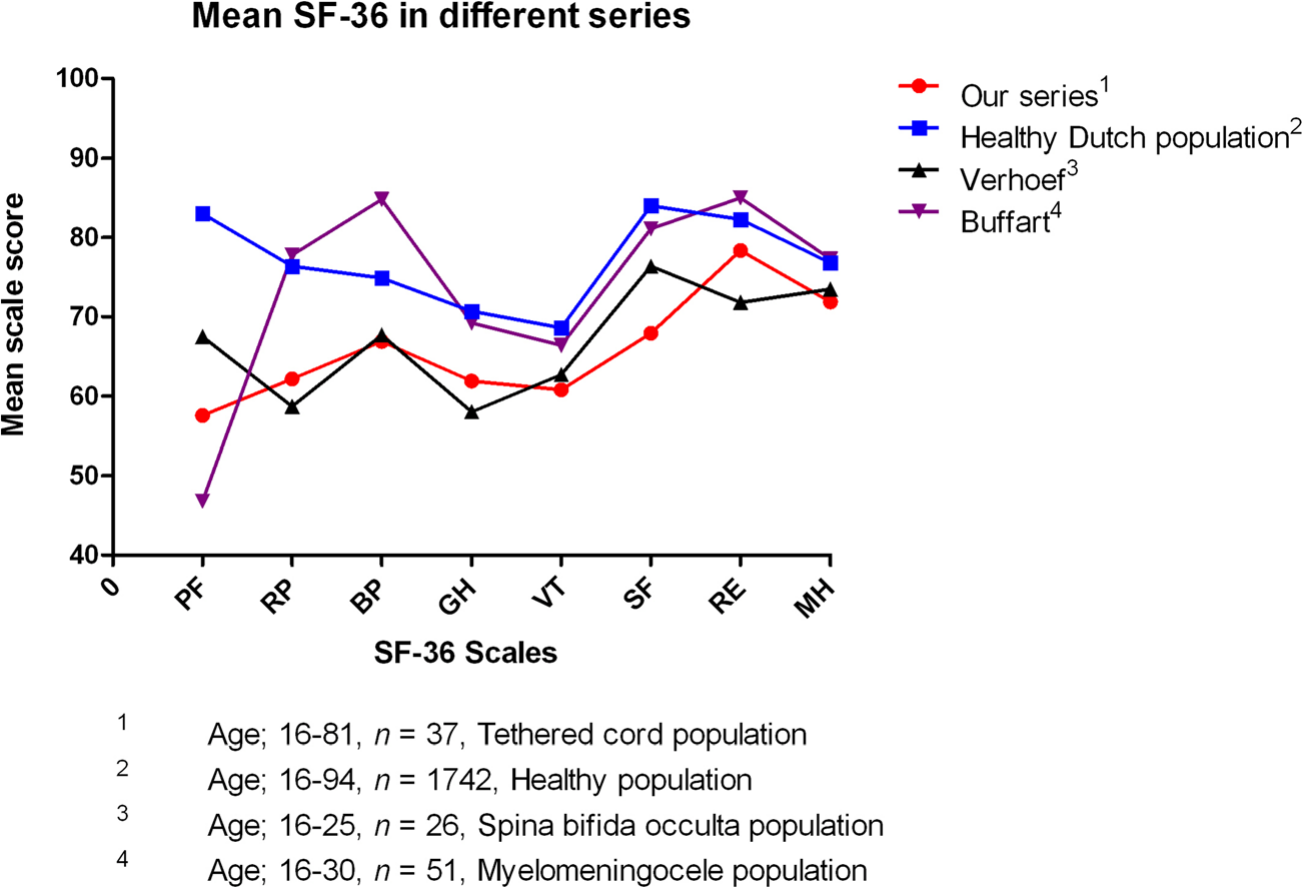
Mean SF-36 scales in different series. Our series and the series of Verhoef et al. showed similar results [40]. The myelomeningocele population from Buffart et al. showed higher scores than our series and was comparable with the results of the healthy Dutch population except on the PF domain [1, 2]. *TC* tethered cord, *PF* physical functioning, *RP* role limitations caused by physical health problems, *BP* bodily pain, *GH* general health perceptions, *VT* vitality, *SF* social functioning, *RE* role limitations caused by emotional problems, *MH* general mental health

In Fig. 6, the myelomeningocele population scored, apart from the physical functioning scale, similar to the healthy Dutch population and even had higher mean scores on three scales (role limitations caused by physical health problems, bodily pain, and role limitations caused by emotional problems). The spina bifida occulta population of Verhoef et al. had the same trend as our series [40]. The physical functioning scale of the myelomeningocele population is lower than in all the other populations.

### Intraoperative neurophysiological monitoring

A mapping protocol was used based on the threshold nerve root stimulation found in the individual patient. Stimulation values below three times threshold were considered functional nerve root structures, and consequently, these structures were never cut. Stimulation values above three times threshold were considered non-functional tethering structures that could be cut. Monitoring using motor evoked potentials was used as a control of the surgical decisions to cut non-functional structures.

The monitoring reinforces the decision-making in surgical detethering based on mapping.

## Discussion

Tethered spinal cord surgery with the use of IONM seems to be long-term effective on the neurological, urological, and pain domains. However, in our study, a high rate of progressive scoliosis was found in the long-term in a group defined by females who underwent surgery at an age at or younger than 12 years.

In Fig. 2, the overall outcome for this group shows a stable long-term outcome in the urological, neurological, and pain domains. However, slight changes in the number of symptomatic patients and the severity of the symptoms, as shown in Table 3 in the appendix, do occur.

A slight decrease in the number of patients with deficits in the neurological and urological domains was observed over time but might be related to better coping of the patients. Pain symptoms decreased primarily postoperatively and remained stable during FU. This indicates that detethering has an immediate and long-term effect on pain. A prospective study would be required to validate these indications.

Scoliosis appeared to be progressive in 11 patients consisting of young females that underwent tethered cord surgery aged 12 years or younger. The question remains whether the progressive scoliosis is due to the tethered cord or caused by other reasons. Several studies, in which patients underwent IONM-assisted tethered cord surgery, do not mention progressive scoliosis as a long-term outcome measure [20, 27, 30, 31, 37, 41]. Few studies present the correlation between tethered cord and the presence of scoliosis [3, 11, 15, 22, 33].

According to Cardoso et al., patients with spinal dysraphism associated with tethering had predisposing factors causing scoliosis [3]. These predisposing factors consisted of unequal growth caused by congenital abnormalities of the spine, instability of the spine caused by missing posterior spinal elements, and paralysis in different severities.

Two earlier studies showed a correlation between scoliosis and tethered spinal cord. McLone et al. focused only on myelomeningoceles. They showed that of patients with curves <50°, 33% had improved and 63% had remained stable 1 year after detethering. At maximum FU of 5 years after detethering, 21% were still improved and 42% were still stable [22]. Reigel et al. followed 262 patients over 20 years to see the effects on progression of scoliosis after tethered cord surgery [22, 33]. They showed that after tethered cord surgery, the progression of scoliosis decreased or reached a plateau when lumbar or sacral surgery had been performed [33]. Besides myelomeningoceles, scoliosis is associated with split cord malformation as well. According to Hilal et al., 40% of patients with split cord malformation will develop scoliosis, especially older children [11].

Tao Yang et al. investigated the clinical presentation of intramedullary neurenteric cysts [43]. They found scoliosis in three patients (23.1%), comparable with our finding of three patients (27.3%) with intramedullary cysts. In three of 11 patients, syringomyelia was present. According to several studies, syringomyelia can be associated with scoliosis [3, 5, 9, 10, 16, 24, 25]. The incidence of scoliosis associated with syringomyelia ranges from 25 to 85% [5, 10, 16].

Jankowski et al. showed that scoliosis is associated with tethered spinal cord and syringomyelia [15]. His patient groups underwent neurosurgery at a young age comparable with our patient group. Jankowski et al. advised that patients with a Cobb angle, <30° should undergo a neurosurgical intervention followed by a period of monitoring for progressive scoliosis. Our findings might suggest that tethered cord surgery may not prevent progression of scoliosis in patients, defined as females that underwent tethered cord surgery before or including the age of 12 years. Like Jankowski et al., we advise close monitoring during FU in order to minimalize the extent of scoliosis progression in these patients.

Both the diagnosis of tethered cord syndrome, in particular split cord malformation, lipomyelomeningocele, and myelomeningocele, and the additional pathology consisting of intramedullary neurenteric cysts and syringomelia indicate a higher chance of obtaining progressive scoliosis. However, this does not explain the high percentage (90.9%) of young females with progressive scoliosis in our sample. When looking at adolescent idiopathic scoliosis, females and males are affected equally. However, progressive scoliosis occurs 10 times more often in females than in males [23]. This might partly explain our high rate of progressive scoliosis in females.

However, when observing the total population in our research with the age of 12 years old or younger, we found that 13 out of 19 (68.4%) females had scoliosis at FU2, and 1 out of 12 (8.3%) males had scoliosis at FU2. Females and males in our sample are therefore not equally affected. It seems there is a predisposition to (progressive) scoliosis in females diagnosed with tethered cord syndrome when taking both diagnosis and comorbidity into account.

Our subgroup analysis of patients with a lipomyelomeningocele showed no deterioration postoperatively.

This may underscore the value of using IONM during high-risk detethering procedures. Concerning the lasting effect of detethering on symptoms as a possible indication of efficacy, our patients with partially resected lipomyelomeningoceles had a PFS of 64.3% after 12.4 years. Other studies with patients with partially resected lipomyelomeningoceles showed a PFS of 52.0% after 10 years [6] and 40.0% after 10 years [29], of which only in the latter study IONM was used. Another study by Pang et al. compared a group of patients with partially resected lipomyelomeningocele and a group with total to near-total resected lipomyelomeningocele [27]. The partially resected group had a PFS of 34.6% after 10.5 years, and the total to near-total group had a 82.8% PFS after 16 years. When only looking at the asymptomatic patients, our series had a PFS of 50% after 11.6 years, while Pang’s series showed a 43.3% PFS in the partially resected group and a 98.4% PFS in the total to near-total resected group. In the total to near-total-resection group, all patients were operated with extensive standard use of IONM. Our series showed a higher PFS when comparing with the other partially resected groups. This might be related to the standard use of IONM in our study. However, Pang’s scorings in the total to near-total resected group, especially asymptomatic patients, would suggest to perform a total to near-total resection in these patients. In a study of Kulkarni et al., a group of asymptomatic patients with lipomyelomeningocele were followed without surgery and showed a PFS of 67% after 9 years [19]. This is even a better result than in our partially resected patients and might be explained by a selection bias due to the small size of our series of asymptomatic lipomyelomeningocele patients.

Although there are different opinions on deciding whether to surgically treat tight filum terminale or not, our series showed an effective surgical outcome with a PFS of 80.0% after 10.5 years with improvements in 40.0% when comparing postoperative with FU2 results.

Drake et al. question whether surgical treatment is doing more good than harm [7, 8]. We propose that when proper criteria are used, consisting of a tethered cord syndrome and the presence of thickened filum in combination with a low-lying conus, improvement can be expected as illustrated in this study.

The quality of life in TC children scored higher than in the healthy Dutch population on every domain except the physical functioning scale. Since the SF-36 is only validated from the age of 16 years old, no strong conclusions can be taken from the results of the TC children group. However, their high score on every scale is still an interesting result. This might be due to the fact that their questionnaire was mostly filled in by their parents. Children themselves also may not completely understand the meaning of their impairments and therefore do not apply their impairments on their quality of life.

The myelomeningocele population scored higher than our series and was even comparable with the scores obtained by the healthy Dutch population. Since symptoms due to myelomeningocele are present from birth, these patients, comparing with tethered spinal cord patients, never knew how it was without their symptoms and therefore might cope better with their symptoms resulting in a higher quality of life.

When comparing with another quality of life study in patients with spina bifida occulta, similar results with our population were observed [40].

Tethered cord surgery has been widely practiced in patients with various forms of spinal dysraphism. The usefulness of IONM during these surgical procedures remains a topic of debate, since its value is difficult to evaluate in an objective manner. A randomized controlled trial will not be feasible to conduct. Most series report on personal experiences and preferences with regard to its use [35]. The prospective cohort study by Pang illustrates the importance of the use of IONM when striving for radical resection of lipomyelomeningoceles. The validation of the use of IONM in tethered cord surgery will grow when it is applied in a standardized fashion and more series will be described. These reports will be valuable in order to define criteria for the use of IONM in tethered cord surgery.

## Conclusion

- The neurological, urological, and pain domains showed stability after detethering in long-term FU in tethered spinal cord patients. This might indicate a lasting effect of surgical detethering with the use of IONM in this series.
- Close monitoring during follow-up might contribute to early recognition of progressive scoliosis, in spite of detethering, in a risk group defined by females who underwent tethered cord surgery under or at the age of 12 years with either lipomyelomeningocele, split cord malformation, or myelomeningocele. Detethering does not appear to protect these patients for progressive scoliosis.

## Appendix

**Table 3:** The changes in outcome, when comparing postoperative with FU2 outcome, per domain per group (HRG, LRG, Redo)

Changes postoperative versus FU2		Neurological						Urological						Orthopedic						Pain					
		HRG (*n* = 26)		LRG (*n* = 24)		Redo (*n* = 10)		HRG (*n* = 26)		LRG (*n* = 24)		Redo (*n* = 10)		HRG (*n* = 25)^a^		LRG (*n* = 24)		Redo (*n* = 10)		HRG (*n* = 26)		LRG (*n* = 24)		Redo (*n* = 10)	
		*N*	%	*N*	%	*N*	%	*N*	%	*N*	%	*N*	%	*N*	%	*N*	%	*N*	%	*N*	%	*N*	%	*N*	%
Improved	1 step	1	3.8	2	8.3	–	–	2	7.7	1	4.2	–	–	–	–	–	–	1	10.0	2	7.7	2	8.3	3	30.0
	2 steps	2	7.7	1	4.2	–	–	2	7.7	1	4.2	–	–	–	–	–	–	–	–	–	–	–	–	–	–
	3 steps	–	–	–	–	–	–	1	3.8	–	–	–	–	–	–	–	–	–	–	–	–	–	–	1	10.0
	Subtotal	3	11.5	3	12.5	–	–	5	19.2	2	8.3	–	–	–	–	–	–	1	10.0	2	7.7	2	8.3	4	40.0
Stable	20	76.9	19	79.2	8	80.0	20	76.9	20	83.3	9	90.0	18	72.0	21	87.5	8	80.0	22	84.6	20	83.3	6	60.0	
Deteriorated	1 step	2	7.7	2	8.3	2	20.0	–	–	1	4.2	–	–	4	16.0	1	4.2	1	10.0	–	–	2	8.3	–	–
	2 steps	1	3.8	–	–	–	–	–	–	–	–	–	–	3	12.0	2	8.3	–	–	2	7.7	–	–	–	–
	3 steps	–	–	–	–	–	–	1	3.8	1	4.2	1	10.0	–	–	–	–	–	–	–	–	–	–	–	–
	Subtotal	3	11.5	2	8.3	2	20.0	1	3.8	2	8.3	1	10.0	7	28.0	3	12.5	1	10.0	2	7.7	2	8.3	–	–

^a^One missing data

The difference between subtotal improvement and the subtotal deteriorations is partly visualized in Fig. 2.

*N* number, *HRG* high-risk group, *LRG* low-risk group, *Redo* redetethering

## Abbreviations

IONM: Intraoperative neurophysiological monitoring
FU: Follow-up
LRG: Low-risk group
HRG: High-risk group
Redo: Redetethering
VAS: Visual analogue scale
MRI: Magnetic resonance imaging
PFS: Progression free survival
TC: Tethered cord
SD: Standard deviation
SF-36: the Medical Outcome Study 36-item Short-Form Health Survey
